# *T*_1_ Relaxation Time in Lungs of Asymptomatic Smokers

**DOI:** 10.1371/journal.pone.0149760

**Published:** 2016-03-09

**Authors:** Daniel F. Alamidi, Simon S. I. Kindvall, Penny L. Hubbard Cristinacce, Deirdre M. McGrath, Simon S. Young, Josephine H. Naish, John C. Waterton, Per Wollmer, Sandra Diaz, Marita Olsson, Paul D. Hockings, Kerstin M. Lagerstrand, Geoffrey J. M. Parker, Lars E. Olsson

**Affiliations:** 1 Department of Radiation Physics, Institute of Clinical Sciences, Sahlgrenska Academy, University of Gothenburg, Gothenburg, Sweden; 2 Department of Medical Physics, Lund University, Translational Sciences, Malmö, Sweden; 3 Centre for Imaging Sciences and Biomedical Imaging Institute, Manchester Academic Health Sciences Centre, University of Manchester, Manchester, United Kingdom; 4 AstraZeneca R&D, Alderley Park, United Kingdom; 5 Department of Translational Medicine, Lund University, Malmö, Sweden; 6 AstraZeneca R&D, Mölndal, Sweden; 7 Medtech West, Chalmers University of Technology, Gothenburg, Sweden; 8 Antaros Medical, BioVenture Hub, Mölndal, Sweden; 9 Bioxydyn Ltd, Manchester, United Kingdom; Telethon Institute for Child Health Research, AUSTRALIA

## Abstract

**Purpose:**

Interest in using *T*_1_ as a potential MRI biomarker of chronic obstructive pulmonary disease (COPD) has recently increased. Since tobacco smoking is the major risk factor for development of COPD, the aim for this study was to examine whether tobacco smoking, pack-years (PY), influenced *T*_1_ of the lung parenchyma in asymptomatic current smokers.

**Materials and Methods:**

Lung *T*_1_ measurements from 35 subjects, 23 never smokers and 12 current smokers were retrospectively analyzed from an institutional review board approved study. All 35 subjects underwent pulmonary function test (PFT) measurements and lung *T*_1,_ with similar *T*_1_ measurement protocols. A backward linear model of *T*_1_ as a function of FEV_1_, FVC, weight, height, age and PY was tested.

**Results:**

A significant correlation between lung *T*_1_ and PY was found with a negative slope of -3.2 ms/year (95% confidence interval [CI] [-5.8, -0.6], p = 0.02), when adjusted for age and height. Lung *T*_1_ shortens with ageing among all subjects, -4.0 ms/year (95%CI [-6.3, -1.7], p = 0.001), and among the never smokers, -3.7 ms/year (95%CI [-6.0, -1.3], p = 0.003).

**Conclusions:**

A correlation between lung *T*_1_ and PY when adjusted for both age and height was found, and *T*_1_ of the lung shortens with ageing. Accordingly, PY and age can be significant confounding factors when *T*_1_ is used as a biomarker in lung MRI studies that must be taken into account to detect underlying patterns of disease.

## Introduction

Chronic obstructive pulmonary disease (COPD) is a complex heterogeneous disease that is a major cause of morbidity and mortality and is considered the third largest cause of death worldwide [1, 2]. There is a major need to develop new treatments for COPD, as no currently available drug therapy suppresses the persistent progression of the disease [3]. Whole-lung spirometric lung function tests are commonly used for characterization of COPD. However, these methods only measure global lung function, resulting in a loss of sensitivity in early/mild disease and pathophysiological abnormalities that may be present in this heterogeneous condition [4, 5]. Improved disease characterization of COPD is therefore needed as it will allow the use of personalised medicine approaches to COPD treatment, an emerging field in which imaging biomarkers are likely to play an important role [6].

In contrast to spirometric lung function tests, regional biomarkers in COPD lungs are sometimes obtained from computed tomography (CT) [7] or single-photon emission computed tomography (SPECT) [8]. The clinical benefits of CT and SPECT for diagnosis of COPD clearly outweigh the potential harmful effects due to ionizing radiation. However for clinical trials, particularly those including a placebo cohort, repeated exposure to ionizing radiation needs to be considered carefully given that there may be no clinical benefit of the examination to the subject. Therefore, non-ionizing radiation imaging techniques are preferred as alternatives in longitudinal assessments in patients with COPD and in therapy monitoring.

Magnetic resonance imaging (MRI) provides attractive biomarkers for assessment of lung disease in clinical trials as it is free from ionizing radiation, minimally invasive and provides regional information [9–11]. Lung MRI has been hampered by the low density of the lung and the fast signal decay due to susceptibility differences between tissue and air in lung parenchyma. Nevertheless, several lung MRI applications have been developed, and interest in MRI of the lungs has recently increased [9–13]. Specifically, it was recently found that the MR specific parameter *T*_1_ relaxation time (subsequently called *T*_1_) was shortened in lung for COPD patients [14]. *T*_1_ measurements of the lung can be used as a read-out to reflect lung function with oxygen-enhanced MRI [12, 15] and to measure partial pressure of oxygen in the alveolar airspaces using hyperpolarised gases [16].

Pulmonary diseases are previously known to influence lung *T*_1_ [17]. Oedema and inflammation lead to an increase in *T*_1_ compared to healthy lung tissue [18]. Shortening of *T*_1_ has been related to fibrosis [19] and emphysema [20]. These factors will also contribute to the *T*_1_ found in COPD patients. However, it is well established that tobacco smoking is a major factor for development of COPD [21, 22], i.e. smokers will be present in COPD cohorts. Smoking results in deposition of particles and coal tar in the lung that induces numerous biological mechanisms responsible for chronic inflammation of the airways and the lung parenchyma and eventually leads to degradation of the lung tissue [23]. Additionally, one could speculate that the presence of tar or other substances [24] that enhance dipolar relaxation in the extracellular tissue water and which accumulate in the lung as a direct consequence of smoking may shorten *T*_1_ directly or the subsequent lung damage may result in a *T*_1_ reduction. However, at present there are no specific data supporting this hypothesis. To our knowledge, the relationship between lung *T*_1_ and tobacco smoke (TS) exposure in healthy subjects has not been previously addressed.

The objective for the present study was to examine whether tobacco smoking influenced *T*_1_ of the lung parenchyma in individuals with no known lung disease. We performed lung *T*_1_ and pulmonary function test (PFT) measurements in asymptomatic current smokers with no diagnosis of lung disease and healthy age-matched never smokers. Healthy smokers were chosen in order to isolate smoking from disease related factors.

## Materials and Methods

### Ethics statement

The study was approved from an institutional review board of the Centre for Imaging Sciences, University of Manchester, UK and the ethical review board of Lund University, Lund, Sweden. All subjects gave written informed consent for examination and data evaluation. The written informed consent in the original study permitted future reanalysis of the data. The work was carried out in accordance with The Code of Ethics of the World Medical Association (Declaration of Helsinki).

### Subjects

Lung *T*_1_ measurements from 35 volunteers, 23 never smokers and 12 current smokers were retrospectively analyzed. Eleven of the never smokers and the smokers were extracted from an existing study from Manchester, United Kingdom, center 1. The other never smokers were from an existing study in Malmö, Sweden, center 2. All 35 subjects underwent lung *T*_1_ and PFT measurements with a similar *T*_1_ measurement sequence. Each volunteer completed a questionnaire before recruitment to assess their suitability for the study. The enrolled subjects had no previous diagnosis of emphysema, bronchitis, chronic asthma, alpha1-antitrypsin deficiency, bronchiectasis or any other chronic lung disease. Any candidate who reported suffering from a cough or chest infection within eight weeks prior to participation was excluded. On the same questionnaire, volunteers recorded details of their smoking history including the smoking of tobacco products other than cigarettes and whether they were regularly exposed to passive smoke.

### Pulmonary function test

Immediately prior, subsequent to or on the day following MRI scanning, standard PFT were carried out to assess forced expiratory volume in 1 s (FEV_1_ (% predicted)) and forced vital capacity (FVC (% predicted)). The measurements were carried out using a computerized spirometer system (Jaeger Oxycon Pro, Hoechberg, Germany) by a trained test administrator according to ATS/ERS standards [25].

### MRI protocol center 1

Imaging was carried out on a 1.5 T-Philips Intera MR system (Philips Medical Systems, Best, Netherlands). In all acquisitions, the q-body coil was used for RF transmission and reception. Throughout the acquisition volunteers were breathing normally, and the imaging was carried out without the use of respiratory or cardiac triggering. A single coronal image slice was positioned at the posterior mediastinum. This slice position gave information on a large area of lung coverage, while avoiding the heart, and was also less likely to be affected by through-plane breathing motion (chest breathing) than more anterior slices. A snapshot FLASH (Fast Low Angle Shot) [26] was used with an initial non-selective inversion pulse. The imaging parameters were: repetition time (TR) 2.2 ms, echo time (TE) 1.0 ms, field of view (FOV) 450 x 450 mm^2^, flip angle (FA) 5°, 64 x 256 matrix (zero filled to 256 x 256) and a slice thickness of 15 mm. In all, 25 inversion times (TI) were used, with an initial TI of 74 ms acquired at intervals of 143 ms, and the measurement was repeated 10 times over a one minute period.

### MRI protocol center 2

Imaging was performed on a 1.5 T-Siemens Magnetom Avanto Fit (Siemens Healthcare, Erlangen, Germany) with a similar approach, slice location and protocol to that used in center 1. Following a non-selective inversion pulse, 16 coronal TIs were acquired with the Snapshot FLASH (21) (TR 3 ms, TE 0.7 ms, FOV 450 x 450 mm^2^, FA 7°, 128 x 64 matrix zero filled to 256 x 256 and a slice thickness of 15 mm) ranging from 107 ms at intervals of 192 ms, during an light inspiration breath hold over 3 seconds.

### Image analysis

Images were registered using techniques defined in [27] to remove respiratory motion in center 1, where free breathing was used, and *T*_1_ was obtained by fitting the Look-Locker equation [28] pixel-by-pixel for the single slice. A region of interest was manually placed on the left and right lungs and was used to calculate the median *T*_1_ value for each subject. The large pulmonary vessels were manually excluded in the quantification. All data analysis was performed using software written in Matlab (MATLAB, The MathWorks Inc., Natick, MA, USA).

### Statistical analysis

First, a potential effect of center (Malmö/Manchester) on *T*_1_ was investigated using a multiple regression analysis adjusted for age, weight, height, FEV_1_ and FVC among the never smokers. The reason for including only the never smokers in this analysis was that never smokers were examined at both centers while all smokers had been examined at a single center. Thereafter, in order to select the most important variables in determining the value of *T*_1_, a backward linear model approach was used. The starting model included FEV_1_, FVC, weight, height, age and pack-years (number of years or equivalent years in which 20 cigarettes a day were smoked, PY) as covariates. Stepwise exclusion of the least significant covariate and refitting of the model was stopped when all remaining covariates showed a significance level of <0.1 with *T*_1_. A simpler model containing only PY and age was also examined, to compare the individual influence of PY and age on *T*_1_. When evaluating the two final models a p-value <0.05 was considered significant. Due to limited sample size the approach taken is exploratory, i.e. no correction for possible model over-fitting was applied. If not stated otherwise, the reported values are given as the mean ± one standard deviation (SD). Analyses were performed using RStudio (version 0.98.507).

## Results

**Fig 1:**
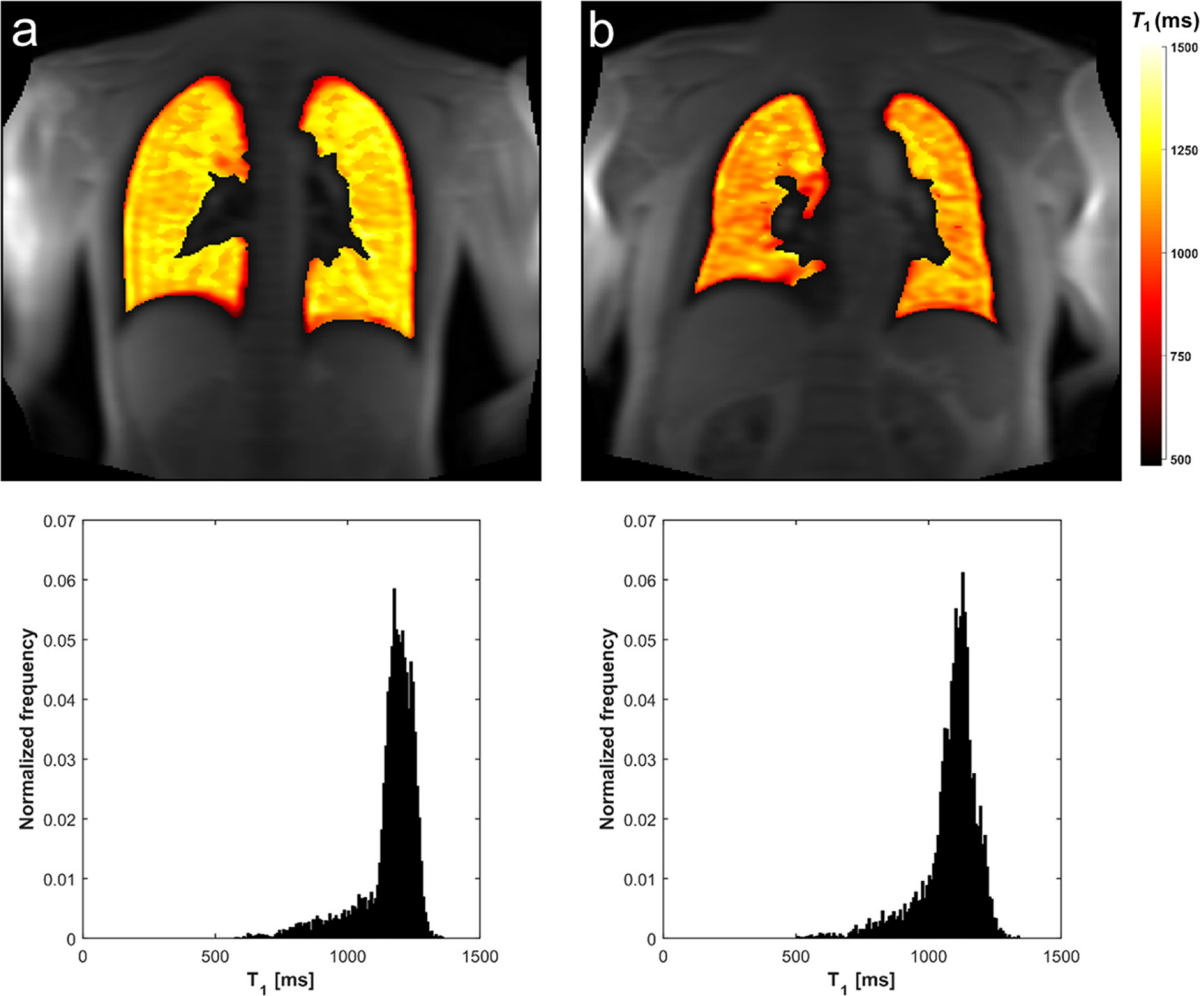
Lung *T*_1_ maps in a young and old never smoker. Representative coronal lung MRI *T*_1_ maps overlaid on a signal intensity image with corresponding normalized *T*_1_ histograms for a 25 years old (a) and a 60 years old (b) never smoker.

**Fig 2:**
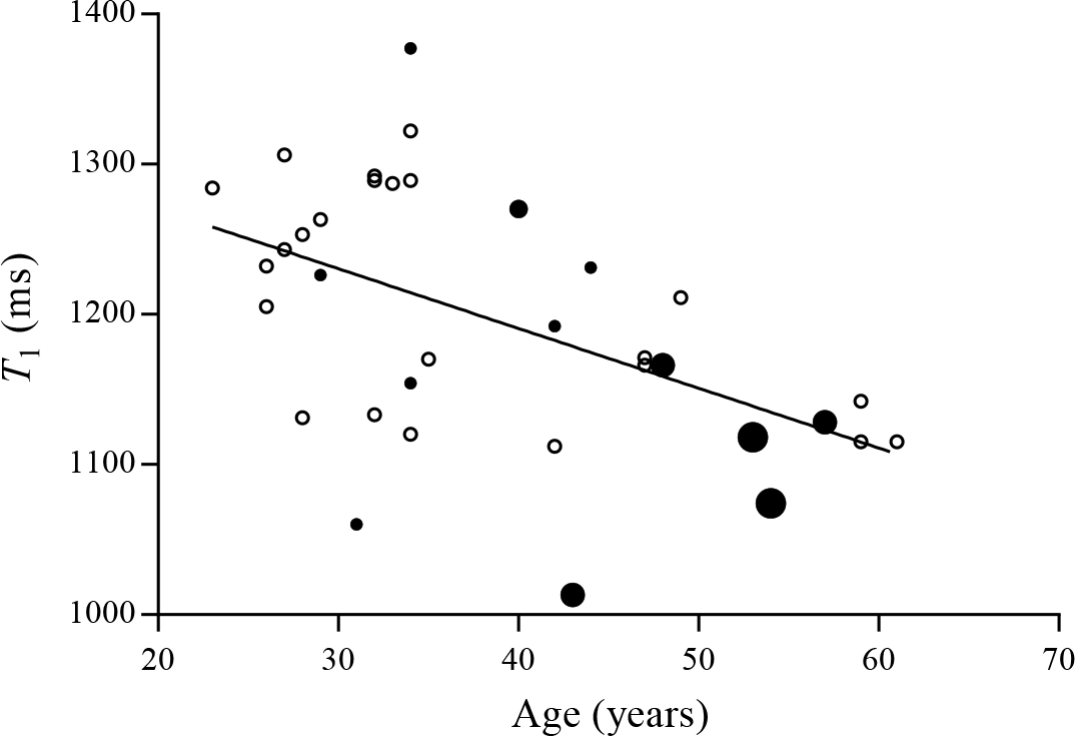
Lung *T*_1_ as a function of age and PY for all subjects. The example line shows the correlation between median lung *T*_1_ and age for smokers (●) and never smokers (○), indicating that lung *T*_1_ shortens with ageing (p<0.01, r = -0.52). The smoking history of the current smokers is visualized with increased size of the dots (• = 1–10 PY, • = 11–20 PY, • = 21–30 PY and • = 31–40 PY).

The MRI examinations were completed in all subjects with diagnostic quality. Representative lung *T*_1_ maps of two subjects with corresponding histograms are provided in Fig 1. The means ± SD of demographic and PFT parameters for all participants are given in Table 1. The current smokers had smoking histories ranging from 2 to 40 PY (mean 16 ± 12 PY) (Fig 2). The never smoking group included an ex-smoker with a smoking history of 2.5 PY. No significant difference on *T*_1_ was found between the centers among the never smokers from the multiple regression analysis (p = 0.35).

**Table 1:** Demographic and pulmonary function data.

	Never smokers center 1	Never smokers center 2	Current smokers center 1
**No. of subjects**	11	12	12
**No. of men**	4	6	6
**Age (y)**	29 ± 4 (23–35)	44 ± 12 (26–61)	43 ±10 (29–60)
**Weight (kg)**	76 ± 14 (61–97)	76 ± 13 (53–104)	77 ± 21 (50–118)
**Height (cm)**	171 ± 12 (150–186)	175 ± 8 (167–188)	173 ± 11 (159–184)
**Smoking index (pack-years)**	0.2 ± 0.8 (0–1.2)	0	16 ± 12 (2–40)
**Pulmonary function measurement**			
**FEV**_**1**_ **(%pred)**	99 ± 20 (69–124)	104 ± 13 (85–130)	102 ± 38 (39–197)
**FVC (%pred)**	112 ± 29 (68–177)	117 ± 13 (100–148)	127 ± 34 (81–187)
**FEV**_**1**_**/FVC**	0.77 ± 0.12 (0.55–0.88)	0.89 ± 0.09 (0.75–1.04)	0.67 ± 0.16 (0.37–0.91)

Data are means ± standard deviations, with ranges in parentheses. Center 1 –Manchester, center 2 –Malmö. ND = no data.

Weight, FEV_1_ and FVC were stepwise excluded in the backward regression procedure for all subjects. The resulting model included PY, age and height as covariates with negative slopes of -3.2 ms/year (95% confidence interval [CI] [-5.8, -0.6], p = 0.02), -2.9 ms/year (95% CI [-5.3, -0.5], p = 0.02) and -2.4 ms/cm (95% CI [-5.0, 0.2], p = 0.07), respectively (Table 2). Excluding height in the simpler model, the slopes of *T*_1_ versus age and PY changed to -3.1 ms/year (95% CI [-5.5, -0.6], p = 0.02) and -2.3 ms/year (95% CI [-4.9, 0.3], p = 0.08), respectively. The negative slope was -4.0 ms/year (95% CI [-6.3, -1.7], p = 0.001) when only age was included in the model with r = -0.52, indicating that lung *T*_1_ shortens with ageing (Fig 2). Among the never smokers, the slope of *T*_1_ as a function of age was found to be -3.7 ms/year (95% CI [-6.0, -1.3], p = 0.003).

**Table 2:** Influence of covariates on *T*_1_ for all subjects (n = 35).

Model	Covariates	Slopes [ms/x]	95% CI	p
1	**Height (cm)**	-2.4	[-5.0, 0.2]	0.07
	**Age (y)**	-2.9	[-5.3, 0.5]	0.02
	**PY (y)**	-3.2	[-5.8, -0.6]	0.02
2	**Age (y)**	-3.1	[-5.5, -0.6]	0.02
	**PY (y)**	-2.3	[-4.9, 0.3]	0.08
3	**Age (y)**	-4.0	[-6.3, -1.7]	0.001
4^a^	**Age (y)**	-3.7	[-6.0, -1.3]	0.003

^a^Among never smokers, n = 23.

## Discussion

Data from this study demonstrate that the association between PY and lung *T*_1_ changes from being significant (p = 0.02) when adjusted for both age and height to being non-significant when adjusted for age only, p = 0.08. There is a significant association between age and *T*_1_ in both final models (adjusted for height and PY, or adjusted for PY only), as well as in the univariate analysis in never smokers. When looking at the PY effect on *T*_1_, it is important to take into account the age of the subjects. Since there is an inherent colinearity between age and PY, it is more likely that subjects with more PY are older. Further investigations in larger cohorts will increase the knowledge of the lung *T*_1_ relationship to PY.

There is evidence showing smoke effect in other imaging studies. Fain *et al*. [29] found that mean ADC values and number of PY were significantly correlated and that relationship remained after adjustment for age with hyperpolarized helium 3 (^3^He) imaging. Additionally, Fain *et al*. also found a strong correlation between mean ADC values and age in both never smokers and healthy smokers. The relationship between ADC, indicating structural changes, and age was explained by microstructural changes in the lung related to the ageing process. ^3^He imaging is a highly sensitive lung imaging technique and the findings with ADC correlations to both PY and age confirms that. Recently, Hamedani *et al*. [30] found functional differences between never smokers, asymptomatic smokers and symptomatic smokers with heterogeneity metrics using ^3^He MR imaging. The smokers recruited in the above mentioned ^3^He imaging studies had similar smoking histories as the smokers in the present study. Taking this knowledge into account, i.e. that structural changes are indeed present in asymptomatic smokers; we might expect that a larger *T*_1_ study would increase the possibility to find a *T*_1_ relationship to PY. Moreover, literature to assist powering a lung MR *T*_1_ study is currently lacking. The results from the present study may be of utility to power future prospective studies to validate these biomarkers.

Recently, we found that lung *T*_1_ correlated to CT density and PFTs in an age-matched COPD cohort study, indicating the potential role of *T*_1_ mapping as a marker of early detection of COPD and emphysema [14]. The observed finding with shortened *T*_1_ in COPD patients was explained by smoking-induced lung pathology, specifically emphysema which was supported by the PFT and CT measurements. No link between lung *T*_1_ and PY was found in the COPD subjects. In the present study, the observed indication with shortened *T*_1_ in the smokers (p = 0.02, adjusted for age and height), therefore, most likely reflects early signs of smoking-induced lung pathology that is not evident from the spirometric measurements.

There are several potential explanations for the lung *T*_1_ relationship to age. In the healthy lung, the blood in the pulmonary circulation is the major source of the assessed lung *T*_1_ at conventional echo times [31]. Blood has a long *T*_1_ (>1000 ms at 1.5 T) [32] and is relatively close to lung *T*_1_ at TEs of the present study (0.7–1 ms). The pulmonary blood volume reduces with age [33] and might therefore explain the shortened lung *T*_1_ with ageing of the lung. The lung tissue of healthy subjects looses its supporting structure with age [34] causing emphysematous changes, which had been shown to shorten lung *T*_1_ [20]. Furthermore, factors such as reduced perfusion and increased macromolecular collagen content are causes that could shorten *T*_1_ in the ageing process of the lung. More accurate models may be constructed with further research incorporating parameters such as hematocrit, oxygenation and other relevant variables to explain the biology behind the *T*_1_ relationship to age. Nevertheless, on the basis of our results, age can be a significant confounding factor when *T*_1_ is used as a biomarker in lung MRI studies that must be taken into account to detect underlying patterns of disease.

There were several limitations with the present study. The small sample size and the study being performed at two centers with slightly different scanning protocols may have introduced an increased uncertainty. However, our multiple regression analysis found that there were no differences between the two centres and it should therefore not affect the analysis of the *T*_1_ measurements. Different breathing protocols were used with free breathing in center 1 and breath hold in light inspiration in center 2. Stadler *et al*. [20] that found a 50 ms difference between full inspiration and expiration, therefore these differences should not be significant for the *T*_1_ measurement. Moreover, the two centers had different TE, 1 ms in center 1 and 0.7 ms in center 2. Measured *T*_1_ depends on what TE is used in the assessment. According to the data from Thriphan *et al*. [31] we should have a systematic 50 ms bias, between the two centers, where center 1 would have longer *T*_1_. We do not believe these small changes affect the conclusions in this study. Another limitation with this study was the two-dimensional MRI protocol that was restricted to one slice and did not cover the whole lung. A multi-slice or three-dimensional protocol would be preferred for improved regional analysis of the smoking-induced effects. Moreover, with regards to the PY measure and the small cohort of smokers, PY is a course measure, as some subjects with very different smoking habit might end up with similar PY values. There was no information on the time between last smoke exposure and imaging.

Regarding these limitations, further prospective studies are desirable to further validate the utility of *T*_1_ mapping in the assessment of healthy smokers. In conclusion, we were able to show a significant relationship between lung *T*_1_ and PY when adjusted for both age and height. Additionally, lung *T*_1_ shortens with increasing age. Thus, PY and age can be significant confounding factors when *T*_1_ is used as a biomarker in lung MRI studies that must be taken into account to detect underlying patterns of disease.

## References

[1] Mannino DM, Watt G, Hole D, Gillis C, Hart C, McConnachie A, et al The natural history of chronic obstructive pulmonary disease. European Respiratory Journal. 2006;27(3):627–43. 10.1183/09031936.06.00024605 16507865

[2] World Health O. World Health Statistics 2008. Geneva, Switzerland: WHO Press; 2008.

[3] Barnes PJ. Chronic obstructive pulmonary disease * 12: New treatments for COPD. Thorax. 2003;58(9):803–8. 12947145 10.1136/thorax.58.9.803 PMC1746809

[4] Bergin C, Müller N, Nichols DM, Lillington G, Hogg JC, Mullen B, et al The diagnosis of emphysema. A computed tomographic-pathologic correlation. The American Review of Respiratory Disease. 1986;133(4):541 3963623 10.1164/arrd.1986.133.4.541

[5] Swanney MP, Ruppel G, Enright PL, Pedersen OF, Crapo RO, Miller MR, et al Using the lower limit of normal for the FEV1/FVC ratio reduces the misclassification of airway obstruction. Thorax. 2008;63(12):1046–51. 10.1136/thx.2008.098483 18786983

[6] Agusti A. The path to personalised medicine in COPD. Thorax. 2014;69(9):857–64. 10.1136/thoraxjnl-2014-205507 24781218

[7] Lynch DA. Imaging of small airways disease and chronic obstructive pulmonary disease. Clinics in chest medicine. 2008;29(1):165–79. 10.1016/j.ccm.2007.11.008 18267190

[8] Jögi J, Ekberg M, Jonson B, Bozovic G, Bajc M. Ventilation/perfusion SPECT in chronic obstructive pulmonary disease: an evaluation by reference to symptoms, spirometric lungfunction and emphysema, as assessed with HRCT. European journal of nuclear medicine and molecular imaging. 2011;38(7):1344–52. 10.1007/s00259-011-1757-5 21365251

[9] Wild JM, Marshall H, Bock M, Schad LR, Jakob PM, Puderbach M, et al MRI of the lung (1/3): methods. Insights into imaging. 2012;3(4):345–53. 10.1007/s13244-012-0176-x 22695952 PMC3481083

[10] Biederer J, Beer M, Hirsch W, Wild J, Fabel M, Puderbach M, et al MRI of the lung (2/3). Why… when… how? Insights into imaging. 2012:1–17. 10.1007/s13244-011-0146-8 PMC3481084 22695944

[11] Biederer J, Mirsadraee S, Beer M, Molinari F, Hintze C, Bauman G, et al MRI of the lung (3/3)—current applications and future perspectives. Insights into imaging. 2012:1–14. 10.1007/s13244-011-0142-z PMC3481076 22695943

[12] Morgan AR, Parker GJM, Roberts C, Buonaccorsi GA, Maguire NC, Cristinacce PLH, et al Feasibility assessment of using oxygen-enhanced magnetic resonance imaging for evaluating the effect of pharmacological treatment in COPD. European Journal of Radiology. 2014;83(11):2093–101. 10.1016/j.ejrad.2014.08.004 25176287

[13] Zhang W-J, Hubbard Cristinacce PL, Bondesson E, Nordenmark LH, Young SS, Liu Y-Z, et al MR Quantitative Equilibrium Signal Mapping: A Reliable Alternative to CT in the Assessment of Emphysema in Patients with Chronic Obstructive Pulmonary Disease. Radiology. 2015. 10.1148/radiol.14132953 25575114

[14] Alamidi DF, Morgan AR, Hubbard Cristinacce PL, Nordenmark LH, Hockings PD, Lagerstrand KM, et al COPD Patients Have Short Lung Magnetic Resonance T1 Relaxation Time. COPD: Journal of Chronic Obstructive Pulmonary Disease. 2015:1–7. 10.3109/15412555.2015.1048851 26488310

[15] Edelman RR, Hatabu H, Tadamura E, Li W, Prasad PV. Noninvasive assessment of regional ventilation in the human lung using oxygen–enhanced magnetic resonance imaging. Nature medicine. 1996;2(11):1236–9. 8898751 10.1038/nm1196-1236

[16] Deninger AJ, Eberle B, Ebert M, Grossmann T, Heil W, Kauczor HU, et al Quantification of Regional Intrapulmonary Oxygen Partial Pressure Evolution during Apnea by 3He MRI. Journal of Magnetic Resonance. 1999;141(2):207–16. 10579944 10.1006/jmre.1999.1902

[17] Stadler A, Stiebellehner L, Jakob PM, Arnold JF, Eisenhuber E, von Katzler I, et al Quantitative and o(2) enhanced MRI of the pathologic lung: findings in emphysema, fibrosis, and cystic fibrosis. International journal of biomedical imaging. 2007;2007:23624 10.1155/2007/23624 17710253 PMC1934944

[18] Bottomley PA, Hardy CJ, Argersinger RE, Allen‐Moore G. A review of 1H nuclear magnetic resonance relaxation in pathology: are T1 and T2 diagnostic? Medical physics. 1987;14(1):1–37. 3031439 10.1118/1.596111

[19] Dasenbrook EC, Lu L, Donnola S, Weaver DE, Gulani V, Jakob PM, et al Normalized T1 Magnetic Resonance Imaging for Assessment of Regional Lung Function in Adult Cystic Fibrosis Patients-A Cross-Sectional Study. PloS one. 2013;8(9):e73286 10.1371/journal.pone.0073286 24086277 PMC3783461

[20] Stadler A, Jakob PM, Griswold M, Stiebellehner L, Barth M, Bankier AA. T1 mapping of the entire lung parenchyma: Influence of respiratory phase and correlation to lung function test results in patients with diffuse lung disease. Magnetic Resonance in Medicine. 2007;59(1):96–101. 10.1002/mrm.21446 18098282

[21] Hanrahan JP, Sherman CB, Bresnitz EA, Emmons KM, Mannino DM. Cigarette smoking and health. American Thoracic Society. American Journal of Respiratory and Critical Care Medicine. 1996;153(2):861–5. 8564146 10.1164/ajrccm.153.2.8564146

[22] Marsh S, Aldington S, Shirtcliffe P, Weatherall M, Beasley R. Smoking and COPD: what really are the risks? European Respiratory Journal. 2006;28(4):883–4. 10.1183/09031936.06.00074806 17012635

[23] Gerhardsson de Verdier M. The Big Three Concept. Proceedings of the American Thoracic Society. 2008;5(8):800–5. 10.1513/pats.200806-058TH 19017732

[24] Kilburn KH. Particles causing lung disease. Environmental health perspectives. 1984;55:97 6376114 10.1289/ehp.845597 PMC1568366

[25] Miller MR, Hankinson J, Brusasco V, Burgos F, Casaburi R, Coates A, et al Standardisation of spirometry. Eur Respir J. 2005;26(2):319–38. 16055882 10.1183/09031936.05.00034805

[26] Jakob PM, Hillenbrand CM, Wang T, Schultz G, Hahn D, Haase A. Rapid quantitative lung (1)H T(1) mapping. Journal of magnetic resonance imaging: JMRI. 2001;14(6):795–9. 11747038 10.1002/jmri.10024

[27] Naish JH, Parker GJM, Beatty PC, Jackson A, Young SS, Waterton JC, et al Improved quantitative dynamic regional oxygen-enhanced pulmonary imaging using image registration. Magnetic Resonance in Medicine. 2005;54(2):464–9. 10.1002/mrm.20570 16032679

[28] Deichmann R, Haase A. Quantification of T1 values by SNAPSHOT-FLASH NMR imaging. Journal of magnetic resonance. 1992;96(3):608–12.

[29] Fain SB, Panth SR, Evans MD, Wentland AL, Holmes JH, Korosec FR, et al Early Emphysematous Changes in Asymptomatic Smokers: Detection with 3He MR Imaging 1. Radiology. 2006;239(3):875–83. 16714465 10.1148/radiol.2393050111

[30] Hamedani H, Kadlecek SJ, Ishii M, Xin Y, Emami K, Han B, et al Alterations of Regional Alveolar Oxygen Tension in Asymptomatic Current Smokers: Assessment with Hyperpolarized 3He MR Imaging. Radiology. 2014. 10.1148/radiol.14132809 25322340

[31] Triphan SMF, Jobst BJ, Breuer FA, Wielpütz MO, Kauczor HU, Biederer J, et al Echo time dependence of observed T1 in the human lung. Journal of Magnetic Resonance Imaging. 2015. 10.1002/jmri.24840 25604043

[32] Mai VM, Knight‐Scott J, Berr SS. Improved visualization of the human lung in 1H MRI using multiple inversion recovery for simultaneous suppression of signal contributions from fat and muscle. Magnetic resonance in medicine. 1999;41(5):866–70. 10332866 10.1002/(sici)1522-2594(199905)41:5<866::aid-mrm2>3.0.co;2-d

[33] Meinel FG, Graef A, Sommer WH, Thierfelder KM, Reiser MF, Johnson TRC. Influence of vascular enhancement, age and gender on pulmonary perfused blood volume quantified by dual-energy-CTPA. European Journal of Radiology. 2013;82(9):1565–70. 10.1016/j.ejrad.2013.04.019 23711422

[34] Janssens J, Pache J, Nicod L. Physiological changes in respiratory function associated with ageing. European Respiratory Journal. 1999;13(1):197–205. 10836348 10.1034/j.1399-3003.1999.13a36.x

